# An Automated Sample Preparation Instrument to Accelerate Positive Blood Cultures Microbial Identification by MALDI-TOF Mass Spectrometry (Vitek^®^MS)

**DOI:** 10.3389/fmicb.2018.00911

**Published:** 2018-05-15

**Authors:** Patrick Broyer, Nadine Perrot, Hervé Rostaing, Jérome Blaze, Frederic Pinston, Gaspard Gervasi, Marie-Hélène Charles, Fabien Dachaud, Jacques Dachaud, Frederic Moulin, Sylvain Cordier, Olivier Dauwalder, Hélène Meugnier, Francois Vandenesch

**Affiliations:** ^1^Innovation Unit, Technology Research Department, bioMérieux, Grenoble, France; ^2^Innovation Unit, Biology Research Department, bioMérieux, La Balme Les Grottes, France; ^3^Innovation Unit, Technology Research Department, bioMérieux, Marcy-l’Étoile, France; ^4^ALCYM, Saône, France; ^5^Centre de Biologie et Pathologie Nord, Institut des Agents Infectieux, Hospices Civils de Lyon – Microbiologie 24/24, Lyon, France; ^6^Centre International de Recherche en Infectiologie, INSERM U1111, Université Claude Bernard Lyon 1, CNRS UMR5308, École Normale Supérieure de Lyon, Université de Lyon, Lyon, France

**Keywords:** blood cultures, MALDI-TOF MS, automation, microorganism identification, filtration

## Abstract

Sepsis is the leading cause of death among patients in intensive care units (ICUs) requiring an early diagnosis to introduce efficient therapeutic intervention. Rapid identification (ID) of a causative pathogen is key to guide directed antimicrobial selection and was recently shown to reduce hospitalization length in ICUs. Direct processing of positive blood cultures by MALDI-TOF MS technology is one of the several currently available tools used to generate rapid microbial ID. However, all recently published protocols are still manual and time consuming, requiring dedicated technician availability and specific strategies for batch processing. We present here a new prototype instrument for automated preparation of Vitek^®^MS slides directly from positive blood culture broth based on an “all-in-one” extraction strip. This bench top instrument was evaluated on 111 and 22 organisms processed using artificially inoculated blood culture bottles in the BacT/ALERT^®^ 3D (SA/SN blood culture bottles) or the BacT/ALERT Virtuo^TM^ system (FA/FN Plus bottles), respectively. Overall, this new preparation station provided reliable and accurate Vitek MS species-level identification of 87% (Gram-negative bacteria = 85%, Gram-positive bacteria = 88%, and yeast = 100%) when used with BacT/ALERT^®^ 3D and of 84% (Gram-negative bacteria = 86%, Gram-positive bacteria = 86%, and yeast = 75%) with Virtuo^®^ instruments, respectively. The prototype was then evaluated in a clinical microbiology laboratory on 102 clinical blood culture bottles and compared to routine laboratory ID procedures. Overall, the correlation of ID on monomicrobial bottles was 83% (Gram-negative bacteria = 89%, Gram-positive bacteria = 79%, and yeast = 78%), demonstrating roughly equivalent performance between manual and automatized extraction methods. This prototype instrument exhibited a high level of performance regardless of bottle type or BacT/ALERT system. Furthermore, blood culture workflow could potentially be improved by converting direct ID of positive blood cultures from a batch-based to real-time and “on-demand” process.

## Introduction

Sepsis and septic shock remain associated with high rates of hospital morbidity and mortality (Wisplinghoff et al., 2004) and share the most often a common cause: a bacteremia or bloodstream infection (BSI). Despite the introduction of alternative diagnostic technologies (van Belkum et al., 2012), blood culture remains the “gold standard” for detecting and isolating the causative microorganism (Dubourg and Raoult, 2015). An inversed temporal correlation was demonstrated between the patient survival and the rapidity of diagnostic of BSI (Valles et al., 2003; Kumar et al., 2006; Rubach and Hanson, 2015). Introduced at the end of 2009, the matrix-assisted laser desorption ionization time-of-flight mass spectrometry (MALDI-TOF MS) is now a common tool in microbiology laboratories for microorganism identification (ID) on solid media allowing significant time savings (Lavigne et al., 2013). However, the ID workflow of positive blood cultures requires 18–48 h of additional time for subculture to provide isolated colonies and delays the initiation of targeted antibiotic therapy. Based on a “house-made” protocol including centrifugation and many manual steps, the direct ID by MALDI-TOF MS of positive blood cultures was associated with an 11.3% increase of patients receiving appropriate antibiotic treatment within 24 h of blood culture positivity in comparison to a control group using MALDI-TOF MS ID on subcultures (Vlek et al., 2012).

To hasten this process, various purification and extraction methods have been developed recently to circumvent subculture step: these are generally manual methods based on abbreviated solid media culture (Verroken et al., 2015; Delport et al., 2016), lysis-centrifugation (Spanu et al., 2012; Foster, 2013), lysis-filtration (Fothergill et al., 2013; Machen et al., 2014), serum separator tubes (Barnini et al., 2015), or some combination of each (Chen et al., 2015). Previous work with lysis-filtration methods showed similar ID results compared with lysis-centrifugation protocols even if the conditions of MALDI-TOF MS ID (e.g., adjustment of cut-off value in case of Bruker MS system) were not directly comparable. However, manual processing by either approach is labor-intensive, costly, exhibits quite acceptable level of performance (closed to 70–80%) with differences between Gram-positive and -negative isolates (Farina et al., 2015), and cannot replace subculture step witch is mandatory for definite ID and antibiotic susceptibility test (AST) results.

Both bioMerieux (Marcy l’Etoile, France) and Bruker (Bremen, Germany) have developed manual research use only (RUO) reagent kits and protocols to facilitate implementation of direct ID from positive blood culture in routine practice. The correct ID performances published are around 80% (Morgenthaler and Kostrzewa, 2015) and 78% (Machen et al., 2014), respectively, for Bruker SepsiTyper^®^ and bioMerieux Vitek^®^MS Blood Culture kits. However, these protocols include several timed washing and extraction steps, often require a dedicated technologist to process positive bottles in continuous flow or by small batches, and thus are also time consuming and labor-intensive. When considering workflow options, most authors process positive blood cultures in batches, thereby reducing the time advantage gained via rapid individual bottle ID results (Loonen et al., 2012).

Recently introduced in microbiology laboratories, the full laboratory automation is now available (Bourbeau and Ledeboer, 2013) to increase productivity and reduce cost per analysis. It also modifies the conventional workflow of microbiological results from a sequential to a continuous process (Dauwalder et al., 2015). Automation could also be employed to address handling of positive blood culture bottles in order to solve issues of limited staffing and labor-intensive protocols. Automation in the microbiology laboratory was initially targeted to handle specimen processing, plate inoculation, incubation, and evaluation of growth via high-definition image analysis (Bourbeau and Swartz, 2009; Favaro et al., 2015). Recently, automated processing and ID of positive blood cultures using intrinsic fluorescence and robotics has been described and evaluated in routine practice (Walsh et al., 2013; Hyman et al., 2016). Similarly, commercial products exist which automate the formic acid (FA) and α-cyano-4-hydroxycinnamic acid (CHCA) matrix dispensing for MALDI-TOF MS analysis (i.e., Bruker Galaxy^®^) or to ensure correct manual spotting and traceability of MALDI-TOF MS target slides (i.e., Copan MALDI-Trace^®^). Although useful for large microbiology laboratories, these products are not practical for small or medium labs due to cost investment and infrastructure modification (Patel, 2016) and could be justified only when the workload is sufficient.

This study describes the development and performance evaluation of an instrument prototype allowing for complete automated processing of positive blood cultures from extraction to target slide spotting to obtain rapid ID of microorganisms with only 2 min of hands-on time. The ID obtained with Vitek^®^MS system using this prototype from seeded and clinical blood cultures was compared with those obtained from colonies recovered from standard subcultures to solid media.

## Materials and Methods

### The Filter Wand Extraction and Spotting Principle

**FIGURE 1:**
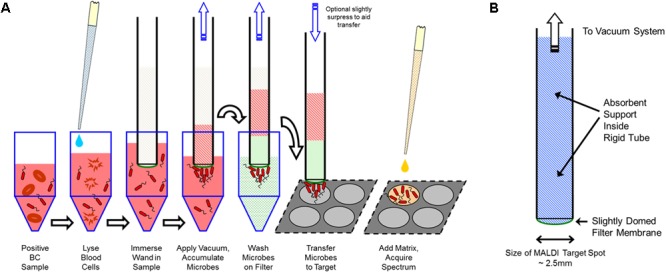
**(A)** Filter wand extraction and spotting concepts. **(B)** Filter wand schematic description (from bioMérieux patent W02013/016211).

The MALDI-TOF MS slide preparation prototype was built around the filter wand (FW) concept, previously patented (Broyer et al., 2015), and an extraction strip pre-filled with lysis and wash buffers. The extraction process of positive blood cultures is based on a selective lysis and filtration principle with the same set of reagents of the Vitek^®^MS Blood Culture kit (bioMérieux, Marcy l’Etoile, France; Fothergill et al., 2013). The extraction and spotting principles are shown in **Figure 1**.

In brief, 1 mL of positive blood culture broth is mixed in the extraction disposable strip containing 0.5 mL of selective lysis buffer (0.6% polyoxyethylene 10 oleoyl ether [Brij97] in 0.4 M [3-(cyclohexylamino)-1-propane sulfonic acid] [CAPS] filtered through a 0.2-μm-pore-size filter, pH 11.7) and incubated for 2 min at room temperature (RT). This step leads to red blood cells’ disruption but keeps microorganisms intact. Then, the FW is immersed into the lysate under vacuum to capture microorganisms onto the filter membrane surface. Under vacuum, the FW is successively transferred to wells containing wash buffers to clean microorganisms bound to the membrane. Finally, the captured microorganisms are transferred to the Vitek^®^MS slide by gently taping the membrane onto the surface of a dedicated slide target spot.

### Performing a BC Sample Preparation Run

#### Rack and Instrument Preparation

**FIGURE 2:**
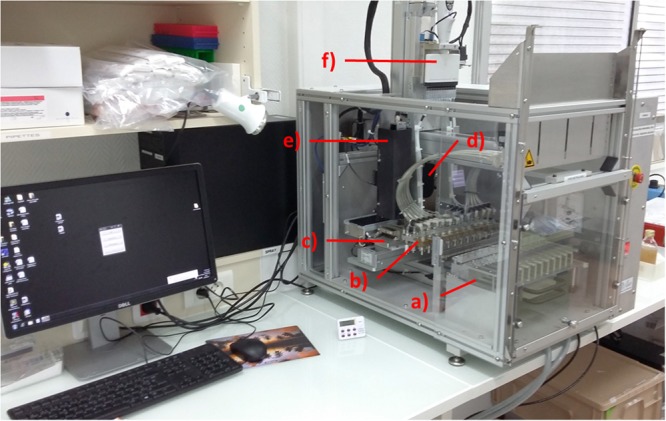
Blood Culture Sample Preparation (BCSP) prototype with the description of the different sub-systems: **(a)** prepared rack; **(b)** pipetting tips and filter wand plate holder; **(c)** MALDI-TOF MS slide holder including formic acid (FA)/α-cyano-4-hydroxycinnamic acid (CHCA) reagents, 96 tips rack and waste; **(d)** Cavro^®^ air displacement pipettor from Tecan for FA and CHCA matrix dispensing; **(e)** MALDI-TOF MS spot imaging sub-system including LED illumination; and **(f)** 12-channel electronic pipette (lysis buffer dispensing).

Only 5 min is needed to prepare the instrument (**Figure 2**) for a full working set of samples. α-Cyano-4-hydroxycinnamic acid (CHCA) matrix and FA tubes, 10-μL carbon tip rack, and Vitek^®^MS slide are loaded in their respective positions (on XY axis holder). CHCA matrix and FA tubes are covered with a specific design sliding cap to prevent solvent evaporation over an 8-h work shift. This cap is opened by the instrument only when a dispensing step is required.

**FIGURE 3:**
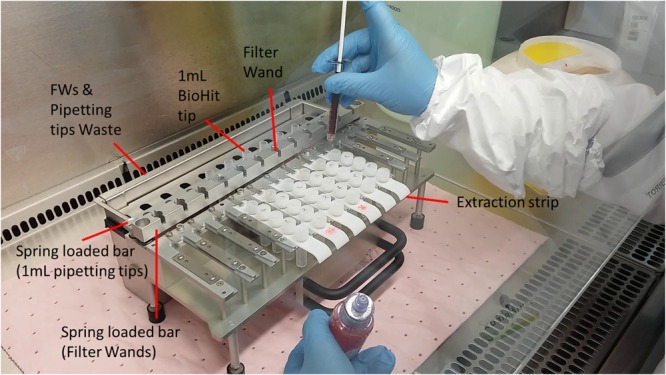
Overview of the preparation of mobile rack with pre-filled extraction strips, 1 mL pipette tips, and assembled filter wands. Only 2–5 min is needed to prepare a run by filling under the class II safety hood 1 mL of positive blood culture in well 1 of each extraction strip.

**FIGURE 4:**
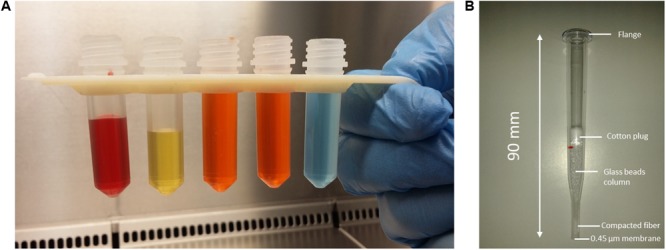
**(A)** Pre-filled tubes snap into a 3D printed support formed the extraction disposable strip. Colored liquids are used to visualize the volume in each tube. From left to right: red = 1 mL of positive BC; yellow = lysis buffer; orange = wash buffer 1 tubes; and blue = wash buffer 2. **(B)** Picture of molded assembled polystyrene crystal filter wand pipette used for prototype evaluation.

The extraction rack (**Figure 3**) is prepared by loading pre-filled extraction strips, pipetting tips, and FWs (**Figure 4**) and by filling the sample well, under biological hood with 1 mL of positive blood culture broth (see section “Design Study”).

A run of a selected number of blood cultures to be extracted and spotted is managed using proprietary software. The software interface controls the extraction modes of extraction, spotting sequences, and the major parameters that could impact final ID (e.g., extraction step duration, vacuum level, number of washes, number of FWs contacts, etc.).

Once the racks are loaded and the run launched, all further steps are performed automatically by the instrument.

#### Extraction Steps

**FIGURE 5:**
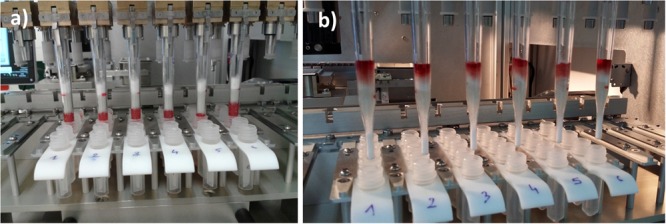
Filter wand’s status **(a)** at the end of the 2-min vacuum aspiration in the lyzed blood cells and **(b)** at the end of wash 2 step showing that all of the aspirated liquids are contained in the filter wand body and blood components are moved far away from the filtration membrane to limit the pollution of MALDI-TOF MS slide with hemoglobin during the spotting step.

If selected, the instrument will take pictures of the blank Vitek^®^MS target slide spots for image subtraction and further analysis at the end of the process. The 1.2-mL pipet tips aspirate 500 μL of lysis buffer from well 2, dispense, and mix in well 1 (20 cycles) with the 1 mL of positive BC. The pipet tips are ejected in the waste rack and replaced with FWs during the 2-min incubation with lysis buffer at RT. At the end of the 2-min lysis step, the FW tips are immersed 4 mm into the lyzed BC solution under controlled vacuum (relative pressure - 600 mbar) for 2 min. The FWs are moved from the lyzed BC to the wash buffer 1 (well 3) for 1 min with alternative Z movements to ensure an efficient removal of the hemoglobin on filtration membrane and on the external wall of the FWs while under vacuum. At the end of this wash step, the FWs are moved to the buffer 1 (well 4) for a 3-min wash in static mode. A final wash step is performed by moving the FWs into buffer 2 (well 5) for 3 min in static mode. To ensure an optimal cleaning of the FWs’ external surface and reduction of hemoglobin peaks in MS spectra (see Supplementary Table S4), the Z level of immersion of FWs is 2 mm deeper in each successive well. Overall, the automated extraction step takes about 11 min. The blood components move into the glass bead pack inside the FW’s body during extraction (**Figure 5a**) in order to get a clean FW membrane at the end of the process (**Figure 5b**). Disposal of pipet tips and FW is checked by an optical sensor placed above the instrument waste receptacles.

#### Filter Wand MALDI-TOF MS Slide Spotting

At the end of the extraction step, the FWs are removed from the wash 2 buffer and a Y arm movement is performed to touch the FW tip on the well edges while maintaining the same vacuum level for 10 s in order to remove excess wash buffer before processing the spotting on the MALDI-TOF MS slide.

**FIGURE 6:**
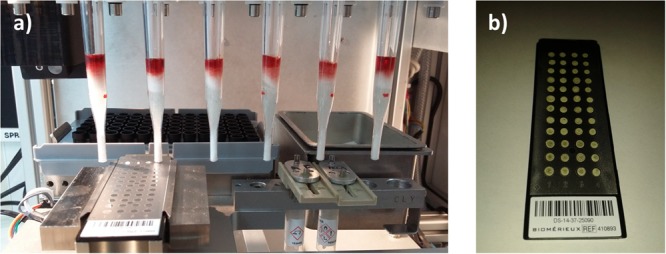
Filter wand (FW) spotting step: **(a)** the FWs are applied on each MALDI-TOF MS spot (three contacts) with a controlled strength and Z level one by one after returning the vacuum level at atmospheric pressure. **(b)** Vitek^®^MS slide aspect at the end of spotting run.

Filter Wand internal pressure is equilibrated to room pressure (by opening three-way valves connected to FW’s aspiration line) and the Y arm is moved to the back of the instrument to start FW spotting onto MALDI-TOF MS target slides. Simultaneous Z arm movements of the FW holder and XY movements of the Vitek^®^MS slide holder are done to successively press each FW’s membrane on two adjacent spots position on the Vitek^®^MS slide (**Figure 6a**). Z displacement is applied with a force of about 0.6 kg on each FW. To improve the robustness of the spotting process (e.g., accept a variability of the FW membrane shape due to the manual assembling steps), three successive contacts are made on each spot per FW.

#### FA and CHCA Matrix Dispensing

In order to avoid dilution of CHCA with the residual water coming from the FW spotting step, a 5-min drying step (at 40°C) of the Vitek^®^MS slide is performed before the addition of 1 μL of matrix using the air displacement pipettor (ADP) successively on four spots. After dispensing CHCA on selected spots, a 3-min incubation at 40°C is performed before imaging (if selected in the software) the prepared Vitek^®^MS slide (**Figure 6b**).

Before analysis, an internal calibrator consisting of a fresh colony of *Escherichia coli* (ATCC 8739) cultured on Colombia Agar + 5% sheep blood agar is manually added to the designated spot on the prepared Vitek^®^MS slide in accordance with the standard Vitek^®^MS procedure.

A 0.5 μL FA droplet is dispensed and evaporated under 40°C incubation during 6 min in the prototype before launching the imaging and CHCA dispensing. The process of dispensing CHCA and FA takes 15 and 18 s, respectively, for four spots using the same 10-μL pipetting tip.

#### MALDI-TOF MS Spot Quality Control by Imaging

**FIGURE 7:**
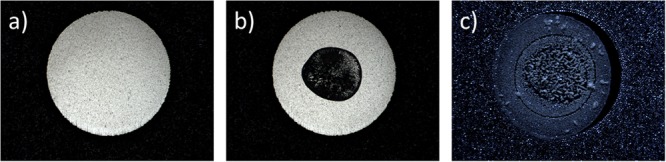
**(a)** MALDI-TOF MS spot before FW application. **(b)** Coaxial illumination to control the biomass transfer after FW application of target slide. **(c)** side Illumination to control the presence of α-cyano-4-hydroxycinnamic acid (CHCA) crystallization with microorganism proteins.

The visual quality of all spots is checked using images acquired with the optical module. Image acquisition could be set at various steps of the run sequence: at the run launch (void spot), after FW biomass transfer, and FA or CHCA matrix dispensing. Using the controlled slide heater, images can be acquired immediately after dispensing to check the presence of FA/CHCA droplets or after a defined drying time to assess spotted biomass and mixing with the FA and CHCA reagents. The quality of the spot is evaluated by checking the presence and the position of a sufficient quantity of biomass relative on the center of the target spot and the correct crystallization of the CHCA mix after drying step. Examples of MALDI-TOF MS spot pictures are provided in **Figure 7**.

## Design Study

### Internal Study With Seeded Blood Culture Bottles in BacT/ALERT^®^ 3D and VIRTUO^TM^ Systems

To determine ID performance, the prototype was tested with the Vitek^®^MS system (bioMérieux, Marcy l’Etoile, France) and paired with BacT/ALERT^®^ 3D system (bioMérieux, Marcy l’Etoile) using standard SA and SN bottles. The same tests were also conducted on the BacT/ALERT^®^ VIRTUO^TM^ (bioMérieux, Marcy l’Etoile, France) using FA Plus and FN Plus bottles. A new detection algorithm is incorporated in the VIRTUO^TM^ system that allows a shorter (4.8 h) time to detection (TTD) of positive bottles (Altun et al., 2016) but the concentration of microorganisms per mL of blood culture broth could be significantly reduced compared with the BacT/ALERT^®^ 3D system. Thus, it was important to determine if FW extraction was sufficient using a lower biomass of microorganism.

### With BacT/ALERT^®^ 3D System

One-hundred-eleven strains representing 60 different species (29 Gram-positive bacteria, 26 Gram-negative bacteria, and five yeast) were selected from our internal strains’ collection (bioMérieux, La Balme, France). The selected strains included the most prevalent organisms associated with bacteremia (Opota et al., 2015), and for the 20 most prevalent, three different strains per species were chosen in order to evaluate the variability of extraction and automatic spotting within the same species. Selected strains were cultured on blood agar plates (BAPs) (bioMérieux, Craponne, France) and incubated 18–24 h in convenient temperature and atmosphere conditions. Serial suspensions and dilutions were prepared and SA bottles (bioMérieux) were inoculated with 30 CFU/mL (1200 CFU per bottle) complemented with 10 mL of blood from healthy donors [Etablissement Français du Sang (EFS), Lyon, France]. Bottles were loaded in BacT/ALERT^®^3D system and signal-positive bottles were retrieved the following day. The slow-growing organisms requiring incubation time longer than 24 h (e.g., yeast) were extracted immediately upon detection by the instrument. All prepared inocula were subcultured to BAP to ensure pure culture. The recovered colonies were also identified using the Vitek^®^MS system.

### With BacT/ALERT VIRTUO^TM^ System

Twenty-two species (nine Gram-negative bacteria, nine Gram-positive bacteria, and four yeasts) corresponding to the most prevalent organisms found in association with bacteremia were selected from our internal strain collection. The same preparation methodology was applied to prepare the inoculated FA Plus bottles (bioMérieux, Marcy l’Etoile) incubated in VIRTUO^TM^ system. To process within an hour after TTD, artificially charged bottles were prepared and loaded in the VIRTUO system at 5 a.m., removed and processed on the Blood Culture Sample Preparation (BCSP) prototype immediately after positivity later that. To evaluate organism density in positive bottles, serial dilutions were prepared of the growth and applied to the surface of BAPs, incubated, and enumerated the next day.

Additionally, for both BacT/ALERT^®^ systems, a negative control consisting of a blood culture bottle with 10 mL of blood was included which was extracted and processed to check for evidence of contamination in extraction reagents, instruments, or disposables.

#### BCSP Rack and Instrument Preparation for Seeded Blood Cultures

The preparation steps were identical for positive blood cultures from either system. The mobile rack was placed under a biological safety hood and loaded with pre-filled extraction strips, 1.2 mL pipetting tips, and FW pipettes. One milliliter was aspirated from each positive bottle and dispensed in well 1 of the extraction strip. Positive bottles that could not be extracted within the 2 h following the TTD were stored at 2–8°C until processing. The instrument rack was loaded with 6–12 extraction strips depending on the number of positive bottles to be processed. For each positive bottle, two replicates were performed and two adjacent spots per FW were done on the target slide leading to four ID results per positive bottle. After FA evaporation, 1 μL of CHCA matrix was automatically dispensed to each MALDI-TOF MS slide position and an image of each spot was recorded (see Supplementary Data).

#### Reference Method and Calculation of ID Score for Seeded Bottles Study

Blood Culture Sample Preparation prototype results from both BacT/Alert^®^ systems were compared to reference ID s obtained from 18–24 h subcultures of positive bottles using the same Vitek^®^MS instrument and duplicate spots (two results) per isolate. ID was generated using Launchpad acquisition (version 1.4.2) software and interpreted with the same knowledge base (2.1.1) used for BCSP prototype prepared slides.

The ID score assigned to the automated method was calculated using the four spots prepared by the system (two bottle extractions duplicates and two spots per FW). No post-treatment manipulation of spectra was done to remove the background noise or modify extraneous peaks.

#### External Study With Clinical Blood Cultures

Patient-based testing was performed at the university hospital of Lyon, France: Hospices Civils de Lyon (HCL) during a 1-month period within the clinical microbiology laboratory where the BCSP prototype was run in parallel with the laboratory standard procedure. Positive patient blood cultures were collected from BacT/ALERT^®^3D (using FA, FN, and PF Plus^®^ bottles) on the primary day shift and processed according to standard laboratory operating procedure including Gram staining, direct examination, subculture on different solid media according to results of the Gram examination, and Vitek MS ID. One milliliter of broth from positive bottles was then manually pipetted in well 1 of the extraction strips previously inserted into the mobile rack.

Gram stain and morphology results were generally available at the time of processing the patient samples. As it improves the ID score for Gram-positive species and notably for *Streptococcus* species, 0.5 μL of FA was systematically added to every spot done with the BCSP prototype whatever was the species (Gram-negative bacteria, Gram-positive bacteria, or yeast).

Only one positive blood culture was tested per patient in order to include the maximum of different patient samples and to cover a larger variability of blood composition and species collection.

At the end of each work shift, the prepared slides from the BCSP prototype were analyzed on the same Vitek^®^MS instrument used to identify microorganisms from the reference subculture (done the next day). The acquisition was done in the HCL laboratory using Vitek MS RUO mode (Acquisition Launchpad 2.9.3 and KB SARAMIS) for direct BCSP prototype. To control for the influence of Vitek^®^MS instrument and KB SARAMIS interpretation, the Vitek^®^MS slides prepared by the BCSP prototype were acquired a second time the following day using our internal Vitek^®^MS instrument in IVD mode (version V2, Acquisition station 1.4.2, KB 2.0.0).

As proposed previously (Idelevich et al., 2014) to calculate the ID performance of the BCSP prototype, if both spots gave the same species ID, the result was considered valid. If discordant species results were given from the two spots, the result was considered incorrect. According to laboratory protocol, isolates of *E. coli* and *Staphylococcus aureus* were not identified by VITEK MS system but by phenotypic standard methods: subculture on URI4 medium and positive indole test (BioRad, Marne la Coquette, France) for *E. coli* and coagulase test directly from positive BC and protein A latex test (bioMérieux, Marcy l’Etoile, France) for *S. aureus* from subcultures.

## Results

### Artificially Inoculated Blood Cultures Incubated in BacT/ALERT^®^ 3D System

Overall 442 Vitek^®^MS results were obtained on 60 species using artificially inoculated blood cultures and BacT/ALERT SA^®^ bottles. Considering all four results per species (four spots made from two FWs’ extraction), a global score of 87% correct ID at species level (confidence score > 70% in Vitek^®^MS) was obtained, with 88% for Gram-positive bacteria (26 species, 196 results), 85% for Gram-negative bacteria (29 species, 210 results), and 100% for yeasts (five species, 36 results).

The global distribution of direct ID results compared with the 18–24 h subculture reference method is summarized in **Table 1**. Detailed results for individual species and specific systems using the direct procedure are presented in Supplementary Tables S1, S2. Comparison of the percentage of ID rate between methods using Chi-square test is given in Supplementary Table S3. The number of strains tested per species is given in Supplementary Table S4.

**Table 1:** Global distribution of identification results with rapid automated method using Blood Culture Sample Preparation (BCSP) prototype and identification by Vitek^®^MS system.

Identification rate (%)			
Type of inoculated microorganism	Reference: 18–48 h subculture + Vitek^®^MS identification	Direct identification from positive blood culture using BCSP prototype with BacT/ALERT 3D, SA bottles, and Vitek^®^MS identification	Direct identification from positive blood culture using BCSP prototype with BacT/ALERT Virtuo, FA Plus bottles, and Vitek^®^MS identification
Gram-negative species	96 % [91–99]%	85% [79–89]%	86% [71–95]%
Gram-positive species	98 % [93–100]%	88% [82–92]%	86% [71–95]%
Yeast species	100 % [81–100%]	100% [90–100]%	75% [48–93]%
Overall ID rate	97.3% [94–99]%	87% [84–90]%	84% [75–91]%

*SA and FA Plus bottles were inoculated with microorganisms belonging to strain collection of the study and were incubated in BacT/ALERT 3D^®^ and BacT/ALERT Virtuo^®^ systems, respectively. Results were compared with reference method based subculture of positive blood culture bottle (COS plate) and identification by Vitek^®^MS system. The results in square brackets are the 95% two-sided exact confidence interval*.

For Gram-positive microorganisms, the percentage of correct ID of some *Streptococcus* species was relatively low. For 12 results, 92% correct ID was achieved for *Streptococcus pneumoniae* and *Streptococcus anginosus*, 83% for *Streptococcus agalactiae*, 75% for *Streptococcus infantarius*, and *Streptococcus oralis* but 67% for *Streptococcus pyogenes*. Despite FA addition, *Propionibacterium acnes* was not identified (eight results, no ID) and *Corynebacterium striatum* was correctly identified for only 8/12 results (67%). *Enterococcus faecium* was correctly identified in 11/12 tests (92%) and 3/4 (75%) for *Staphylococcus lugdunensis*. No discordant results were obtained for the Gram-positive group. The negative control bottles with blood only provided no ID, as expected.

For the Gram-negative group, the main ID difficulties occurred with *Stenotrophomonas maltophilia* (four results, one no ID), *Haemophilus haemolyticus* (eight results, seven no ID, one discordant result wrongly identified as *Listeria grayi*/*S. aureus*), *Neisseria meningitidis* (16 results, 10 no ID), and *Neisseria gonorrhoeae* (16 results, 10 no ID, two discordant results identified as *Corynebacterium mucosalis*). All the others Gram-negative species were correctly identified at species level (92–100%). No results were obtained for *Campylobacter jejuni* which failed to growth in BacT/ALERT SA^®^ bottles.

All five *Candida species* tested were correctly identified (12/12 for *Candida albicans, Candida tropicalis*, and *Candida glabrata*, 4/4 for *Candida parapsilosis* and *Candida krusei*).

The corresponding reference IDs from isolated colonies which were obtained with the same Vitek^®^MS instrument with the same database (i.e., KB), led to an overall correct ID of 97.3% at species level, with 2 no ID results on *Enterobacter cloacae* and *Streptococcus intermedius*, three discordant results on *H. haemolyticus* identified as *H. influenza*, and one discordant result on *S. intermedius* identified as *Streptococcus contellatus*.

Overall, these results demonstrate that the BCSP prototype instrument and protocol used in association with Vitek^®^MS system provided robust ID results for the large majority of the species and for the most prevalent species in sepsis.

### Artificially Inoculated Blood Culture Incubated in the New BacT/ALERT VIRTUO System

The range of VIRTUO TTD results for this study was between 8–10 (*E. coli*) and 36 h (*C. parapsilosis*) and the delay between the TTD and the initiation of the BCSP prototype process was below 1 h (min = 18 min, max = 60 min, mean value = 36 min). Four positive FA plus bottles were extracted with longer delay (120–450 min) that could have led to an increased concentration of the microorganisms (data results in Supplementary Tables S1, S2).

Overall, 88 Vitek^®^MS results from 22 strains were obtained. A global score of 84% correct ID at species level was obtained corresponding to 86% for Gram-positive microorganisms (36 results, nine species), 86% for Gram-negative bacteria (36 results, nine species), and 75% for yeasts (16 results, four species).

Difficulties of ID were encountered with *Staphylococcus epidermidis* (four results, three no ID), *E. cloacae/Enterobacter asburiae* (four results, three no ID), and *C. albican*s (four results, three no ID). One discordant result was obtained for *Enterobacter aerogenes* identified as *E. coli*. Regarding the spot position, this discordant is possibly due to cross contamination during manual spotting of the *E. coli* calibrator on the Vitek^®^MS slide.

The range of organism load in the positive bottles from the Virtuo system ranged between 7 × 10^5^ CFU/mL (*C. tropicalis*) and 2.2 × 10^9^ CFU/mL (*Citrobacter koseri*) with a mean value of 7.06 × 10^8^ CFU/mL. Those ranges fell within the limit of detection for MALDI-TOF MS (10^5^ CFU/ spot), so organism load should not have been an issue.

### Results Using Clinical Blood Cultures

A total of 103 bottles from 102 patients were included in this study, corresponding to 97 monomicrobial, five polymicrobial, and one false-positive blood culture bottles processed by both BCSP prototype and by the standard procedure. The false-positive bottle, which was excluded from the study, was flagged positive by BacT/ALERT^®^3D but led to no visible microorganisms on Gram stain and no growth on subculture after 10 days of incubation. Of the 103 positive bottles, 100 were identified by standard procedure (77 by MALDI-TOF MS). It is important to note that the study included both adult and pediatric patients with a range of associated blood volumes (4% of bottles with blood volume > 10 mL, 26% of bottles with the recommended 10 mL volume, 42% with 5 mL, and 28% with <5 mL).

The 102 processed positive BCs included 53 aerobic, 30 anaerobic, and 19 pediatric bottles. From the 97 monomicrobial positive bottles, 51 Gram-positive bacteria, 34 Gram-negative bacteria, and 12 yeasts were recovered. Overall 83% of monomicrobial positive blood cultures were correctly identified at species level (confidence score > 60% with Vitek^®^MS IVD version) using the BCSP prototype protocol compared to the standard ID procedure used in the laboratory. This included 89% of Gram-negative bacteria (33 bottles), 79% of Gram-positive bacteria (49 bottles), and 78% of yeasts (nine bottles) were correctly identified (**Table 2**).

**Table 2:** Direct identification versus standard procedure from clinical blood cultures (BCs) in routine practice: Gram-negative bacteria, Gram-positive, and yeast.

ID obtained by hospital standard procedure (24–48 h)		« BC Prep Station » + VitekMS (1 h)
Identified microorganisms	Isolate distribution – (%)	Number of correct IDs on total identified bottles – (%)
Gram negative bacilli	36%	59/66 – (89%) [79–96]%
*Escherichia coli*	14%	26/26 – (100%)
*Proteus mirabilis*	4%	8/8 – (100%)
*Enterobacter cloacae*/*asburiae*	3%	4/6 – (67%)
*Klebsiella pneumoniae*	3%	6/6 – (100%)
*Acinetobacter baumannii*	2%	4/4 – (100%)
*Klebsiella oxytoca*	2%	4/4 – (100%)
*Pseudomonas aeruginosa*	2%	3/4 – (75%)
*Fusobacterium nucleatum*	1%	0/2 – (0%)
*Moraxella osloensis*	1%	2/2 – (100%)
*Ochrobactrum anthropi*	1%	2/2 – (100%)
*Pseudomonas putida*	1%	0/2 – (0%)
Gram positive		79/100 (79%) [70–87]%
Gram-positive bacilli	1%	0/2 – (0%)
*Propionibacterium acnes*	1%	0/2 – (0%)
Gram-positive cocci	53%	79/98 – (81%)
*Staphylococcus epidermidis*	24%	37/44 – (84%)
*Staphylococcus aureus*	8%	13/14 – (93%)
*Enterococcus faecalis*	4%	7/8 – (88%)
*Staphylococcus capitis*	3%	5/6 – (83%)
*Micrococcus luteus/lylae*	2%	2/4 – (50%)
*Staphylococcus warneri*	2%	3/4 – (75%)
*Streptococcus constellatus*	2%	1/4 – (25%)
*Staphylococcus haemolyticus*	1%	2/2 – (100%)
*Staphylococcus hominis*	1%	1/2 – (50%)
*Streptococcus agalactiae*	1%	2/2 – (100%)
*Streptococcus dysgalactiae*	1%	2/2 – (100%)
*Streptococcus gallolyticus*	1%	0/2 – (0%)
*Streptococcus mitis/oralis*	1%	2/2 – (100%)
*Streptococcus pneumoniae*	1%	2/2 – (100%)
Yeast	10%	14/18 – (78%) [52–94]%
*Candida albicans*	3%	5/6 – (83%)
*Candida krusei*	1%	2/2 – (100%)
*Candida lusitaniae*	2%	3/4 – (75%)
*Candida parapsilosis*	1%	2/2 – (100%)
*Candida tropicalis*	1%	0/2 – (0%)
*Candida utilis*	1%	2/2 – (100%)
Total	100%	152/184 – (83%) [76–88]%

Focusing on pediatric monomicrobial bottles, the overall ID rate was 94% (34 results, eight species) including 100% of Gram-negative bacilli (four results, three species), 91% of Gram-positive cocci (11 results, three species), and 100% of yeast (two results, two species). There was one discordant result for *S. epidermidis* which was identified as *Mycobacterium fortuitum* (**Table 3**).

**Table 3:** Not identified species and discordant results.

Sample ID	Blood culture bottle type	Direct Gram examination result	Reference Identification (standard procedure based on Vitek^®^MS system)	Vite^®^kMS results on no ID/misidentified clinical blood culture bottles (direct identification from positive blood culture using Blood Culture Sample Preparation (BCSP) prototype and Vitek^®^MS identification)
008	FA	GPC	*Micrococcus luteus/lylae*	No ID
011	FN	GPC	*Streptococcus constellatus*	No ID and DSC
015	FN	GPC	*Staphylococcus epidermidis*	No ID
024	FA	GPC	*Staphylococcus epidermidis*	No ID
027	FN	GPB	*Propionibacterium acnes*	No ID
039	FN	CPC	*Staphylococcus hominis*	No ID
044	FN	GNB	*Pseudomonas aeruginosa*	*P. aeruginosa*/*Bacteroides megaterium*
053	FN	GPC	*Streptococcus gallolyticus*	No ID and DS*C*
069	FA	GNB	*Pseudomonas putida*	No ID
072T	FA	Yeast	*Candida lusitaniae*	No ID
076	FA	GPC	*Staphylococcus epidermidis*	*S. hominis*
090	FN	GNB	*Fusobacterium nucleatum*	*V. parahaemolyticus/ L. monocytogenes*
099	FN	GNB	*Enterobacter cloacae/asburiae*	No ID
103T	FA	Yeast	*Candida tropicalis*	*C. albicans*

*FA, aerobic FA BacT/ALERT bottle incubated in a BacT/ALERT 3D instrument; FN, anaerobic FN BacT/ALERT bottle incubated in a BacT/ALERT 3D instrument; GPC, Gram-positive cocci; GNB, Gram-negative bacilli; GPC, Gram-positive bacilli; No ID, no microorganism identification; DSC, discrepant microorganism identification with the reference.*

The BCSP prototype provided ID of one of two species recovered from subcultures of the five polymicrobial bottles recognized in the study (**Table 4**).

**Table 4:** Polymicrobial results.

Reference identification mixed BC	Direct identification from positive blood culture using Blood Culture Sample Preparation (BCSP) prototype and Vite^®^kMS identification
*Psychrobacter* sp./*Staphylococcus epidermidis*	*Psychrobacter phenylpyr*
*Enterococcus faecalis*/*Acinetobacter baumannii*	*E. faecalis*
*Bacterioides fragilis*/*Staphylococcus hominis*	*B. fragilis*
*S. hominis*/*S. epidermidis*	*S. hominis*/*S. epidermidis^∗^*
*Escherichia coli*/*Candida glabrata*	*E. coli*

*^∗^The first filter wand has identified as *S. hominis* and the second filter wand has identified S. epidermidis.*

## Discussion

During the last decade, integration of the MALDI-TOF MS ID technology in the routine microbiology workflow and advances in automation technologies have led to changes in laboratory organization to reduce time to obtain microorganism ID and to improve laboratories’ productivity with reduced human resources due to health care system budget constraints. At this time, the sepsis diagnosis remains notably based on the positivity of blood cultures and a clear-cut relation between the mortality and the rapidity of administration of effective antimicrobial therapy was demonstrated (Kumar et al., 2009). In order to reduce this last step, different methodologies have been introduced to reduce time to obtain microorganism IDs directly on blood culture bottles and are based on two technologies. The first one is based on different homemade or commercialized manual protocols to directly perform the ID of positive blood culture by MALDI-TOF MS without plate subcultures (Farina et al., 2015; Morgenthaler and Kostrzewa, 2015). Despite the development of rapid manual methods intended to eliminate the need for subculture, limitations remain in implementation due to the high cost of instrumentation and reagents to lack of staffing or technical skills. These constraints led to batch processing of positive blood culture bottles thus delaying pathogen(s) ID and clinically actionable results. Further rapid extraction methods focus only on microorganisms pelleted from positive blood culture requiring additional manual steps to finalize the preparation of a MALDI-TOF MS slide. The second approach is based on molecular syndromic tests performed directly on positive blood samples. Different instruments or methodologies (FilmArray^®^, ePlex^®^, Accelerate Pheno^®^, Verigene^®^, etc.) are used and included or not the detection of some genes encoding antibiotic resistances or accelerated ASTs (Siu et al., 2015; Salimnia et al., 2016; Hogan et al., 2017; Marschal et al., 2017; Surgers et al., 2017). Despites high level of performance for ID (>95% for the most of them), they are limited to the detection of microorganisms included in their panel corresponding to 80–90% of the currently isolated microorganisms in a university hospital. As reported by Rand and Delano (2014) in a comparative study on 161 positive blood cultures including 10 polymicrobial entities, FilmArray BCID identified 132/133 (99%) microorganisms included in its panel corresponding to 132/161 (82%) of the total microorganisms whereas direct ID by VITEK MS instrument using the VITEK MS kit allow the ID of 142/161 (88%) microorganisms for 10$/test including lab technician processing time versus 120$ per FilmArray pouch.

The BCSP prototype addresses these concerns and provides full automation of slide preparation directly from positive blood cultures in less than 25 min. This bench top instrument does not require additional technical skill and requires only 5 min hands-on time with ready-to-use extraction strip reagents. The instrument requires minimal preparation to launch, and does not require decontamination concomitant with the use of the disposable FW. As a result, the BCSP prototype could be used during all shifts to accelerate positive blood culture ID and implementation of appropriate therapy as previously described (Vlek et al., 2014). Recently, a large meta analyze of 31 studies (5920 patients) focused on the clinical impact of rapid ID tools directly performed on positive blood cultures (syndromic molecular test (20 studies), FISH (six studies), and MALDI-TOF MS (four studies) demonstrated a mortality risk reduction, a decrease of 2.48 days of hospitalization duration, and a decrease of 5 h of the time before convenient antimicrobial therapies in combination of antibiotic stewardship (Timbrook et al., 2017). Another pediatric study comparing rapid ID of positive blood culture by FilmArray BCIC panel associated with stewardship decision support versus conventional practice associating subculture of positive blood culture and isolated colony ID by MALDI-TOF MS also demonstrated a decrease of the duration before efficient antibiotic therapies from 60 to 27%, a reduction of inutile antimicrobial treatments, and an adaptation of antimicrobial therapies after BCID result in 73% of the case (Messacar et al., 2016). Thus, these results underline that rapid ID methods performed directly on positive blood culture sample have many impact on the treatment, mortality, and length of hospitalization and should be performed by the microbiological lab.

Performances of this prototype were evaluated with a large and relevant panel of microorganisms using artificially inoculated bottles as well as clinical blood cultures and compared with reference ID methods. Associated with Vitek^®^MS system, the BCSP prototype provides accurate and rapid ID of the microorganisms growing in positive blood cultures. Further, the instrument could be paired with either BacT/ALERT 3D or VIRTUO^TM^ blood culture systems using standard SA/SN or FA/FN Plus resin bottles.

### Identification Results and Standardization of MALDI-TOF MS Slides’ Preparation

An overall performance for the BCSP prototype of 87% correct ID established using artificially inoculated bottles (BacT/ALERT^®^3D system) compared with a 97.3% correct ID using reference standard subcultures obtained the next day suggests that the automated system could provide rapid results for the large majority of positive blood cultures obtained in the clinical microbiology laboratory. The large diversity of organisms covered by the study suggests that the selected extraction and spotting parameters were generic enough to provide reliable sample preparation for the majority of expected isolates. However, some fastidious organisms like *N. meningitidis* and *P. acnes* were not identified. These two species were also not identified in a previous report (Opota et al., 2015) using the same lysis buffer formulation. We have confirmed that these results were due to the lytic action of the buffer leading to bacterial loss during filtering step (data not shown).

When the results of only one of the duplicate spots generated by the FW was considered (only if there was no discrepancy between spots duplicates), the overall correct ID increased to 91% (222 results, 60 species). This included 96% (96 results, 25 species), 85% (108 results, 30 species,) and 100% (18 results, five species) for Gram-positive bacteria, Gram-negative bacteria, and yeasts, respectively.

An overall ID rate of 84% was obtained with the BacT/ALERT VIRTUO^TM^ system when positive blood culture bottles were processed immediately after TTD. This was comparable to results obtained with BacT/ALERT^®^ 3D system demonstrating that the concept works well with a lower concentration of microorganisms per bottles at the time of positivity. Two minutes of aspiration with the FW in the lyzed broth appears to be sufficient to capture and concentrate the required biomass needed for MALDI-TOF MS ID (10^6^ CFU/mL; Croxatto et al., 2012).

Compared to manual rapid methods which provided a reduced ID rate percentage for Gram-positive than for Gram-negative (about 14% fewer with SepsiTyper kit, Morgenthaler and Kostrzewa, 2015), the association of BCSP prototype with Vitek^®^MS did not show a disparity for Gram-positive and Gram-negative species. Possible reasons for this observation are that the systematic addition of 1 μL of FA before the addition of CHCA matrix and better control of FA and CHCA matrix evaporation using the heater block provides for improved Gram-positive ID.

Additionally, in some studies, there were adjustments secondary to the “log-score cut-off” threshold retained for correct species ID determination for the Bruker BioTyper^®^ system. Decreased threshold values from 1.9 to >1.5 were used to improve ID rates with an overall correct species ID average of 75% (Morgenthaler and Kostrzewa, 2015). We hypothesis that these decreases in threshold adjustment, when using positive blood cultures, can be avoided by using a high standardized method for extraction as well as MALDI slide preparation. Thanks to our BCSP prototype, the Vitek^®^MS did not require adjustment of threshold and demonstrated a satisfactory ID rate directly from positive BC by delivering ready to use MALDI slides with a minimal human hand processing. In addition, the removal of residual hemoglobin appears to be a key point for correct ID. This could be due to the fact that reference databases were constructed using MS spectra acquired from pure colonies isolated from solid media. By using the FW and an automated washing sequence, the risk of false or no ID results related to interfering hemoglobin peaks (from alpha and beta chains, peaks near 15, 7.5, and 3.7 kDa (Vestal, 2016) especially with hemolytic species (e.g., *S. pyogenes*) was reduced. Examples of MS spectra and corresponding ID are given in Supplementary Figure S1 showing the importance of hemoglobin peaks when the washing is insufficient compared to improved wash procedure during FW extraction.

### Clinical Performances Using BCSP Prototype With Vitek^®^MS IVD

Using positive blood cultures from patients, a correct ID of 89 and 79% for Gram-negative and Gram-positive species was achieved confirming the performance of this system in a clinical setting. These results were close to those described in published manual in-house methods and were established with systematic addition of FA. Further, they corroborated our internal data showing improved ID rate for Gram-positive species and notably on *Streptococcus* genus.

In most cases, the results were associated to “no ID or insufficient peaks.” The four discordant cases were *P. aeruginosa* identified as *P. aeruginosa*/*Bacillus megaterium, S. epidermidis* identified as *Staphylococcus hominis*; *Fusobacterium nucleatum* identified as *Vibrio parahaemolyticus*/*Listeria monocytogenes*; and *C. tropicalis* identified as *C. albicans*. Seventy-eight percent of positive fungal blood culture bottles were also correctly identified with one discordant result (*C. tropicalis* identified as *C. albicans*, **Table 3**).

The use of image analysis with the BCSP prototype showed that about 30% of “no ID results” were related to an insufficient quantity of biomass transferred from the FW to the target spot. The lack of biomass transfer reproducibility could be attributed to the variability of the manually assembled FW disposable that sometimes generated a flat or concave membrane shape. The assembly process could be improved by the development of a thermal welding tool that would produce reproducible domed shape membrane at the end of the FWs. This would also explain why it was important, at this development stage, to perform two FW and two successive spots from the same FW, to determine the variability of the disposable.

The performance of the BCSP prototype for correct yeast ID was 78% which is in line with the published performance of the SepsiTyper kit (50–100% correct ID, Morgenthaler and Kostrzewa, 2015) but lower than that reported by Fothergill et al. (2013) where a 94.1% correct ID for yeast (*n* = 17) was achieved using the lysis-filtration protocol. Moreover, Idelevich et al. (2014) found 62.5% correct ID (*n* = 24) when using the Sepsityper protocol without prior washing steps but Yan et al. (2011) showed 100% correct ID on clinical yeast positive BC (*n* = 42) by adding two washing steps prior to using the SepsiTyper protocol. This would emphasize the critical impact of washing steps to remove extraneous peaks from MS spectra on ID performance (see example of MS spectra in Supplementary Data).

### Improvement of Turnaround Time and Routine Workflow Implementation

The prototype BCSP prototype instrument provides automated processing with controlled duration of all the steps (extraction, spotting, drying slide, FA, and CHCA addition), thereby reducing the variability of the entire process. This achieves standardization of the slide preparation leading to minimal noise background in MS spectra and robust ID rate.

We estimate from our internal study with seeded bottles that the BCSP prototype (on its current 12 position extraction strip format) is able to process up to 96 positive blood culture per work shift (8 runs × 12 extractions) with minimal hands-on time. When testing this prototype in a clinical laboratory setting, the prototype demonstrated the versatility to process variable batch sizes (from 1 to 10 positive blood cultures) at different times of the working shift (8 a.m. to 5 p.m.) without disturbance of the routine workflow.

## Conclusion

This prototype (i) allows a total automation of these pre analytical steps requiring less than 5 min of lab technician time to start the process; (ii) could be performed “on demand” as well as “small batches” of blood culture; (iii) needs less than25 min to prepare a MALDI-TOF MS slide for ID directly from a positive blood culture; (iv) exhibited high level of performance in line with the best results of previously published papers (Martiny et al., 2012; Fothergill et al., 2013); and (v) could be integrated with the MYLA/VITEK MS software allowing an high level of traceability and an facility of use compatible with an 24/7 service. This concept could provide an alternative approach to improve blood culture management in microbiology laboratories without added labor or workflow adjustment and at any time. In the veterinary and food microbiology sector, it could also shorten the time to obtain result and thus have positive economical and public health impact in a « one Health » perspective.

## Ethics Statement

This study was approved by the Ethics committee of HCL Lyon, France. All subjects provided written informed consent before their inclusion in the study.

## Author Contributions

PB conceived the study and the instrument concept and participated in performance evaluation. GG assisted in study conception. HR, JB, and FP conceived and developed the sub-systems incorporated into the instrument and disposables. FD, JD, FM, and SC developed the instrument prototype. NP and M-HC performed all experiments to establish the identification performances. OD, HM, and FV conceived and supported the clinical study in hospital. PB and OD wrote the manuscript. All authors read and approved the final manuscript.

## Conflict of Interest Statement

OD, FM, and FV from Hospices Civils de Lyon received funding (contract number IPM 15712) from bioMérieux to collect patients samples during the evaluation of the prototype at hospital. The other authors declare that the research was conducted in the absence of any commercial or financial relationships that could be construed as a potential conflict of interest.
