# Supplementary material for: An Automated Sample Preparation Instrument to Accelerate Positive Blood Cultures Microbial Identification by MALDI-TOF Mass Spectrometry (Vitek®MS)
Source: Front Microbiol. 2018 May 15;9:911. doi: 10.3389/fmicb.2018.00911 (PMC5962758; doi:10.3389/fmicb.2018.00911)
Supplement: Supplementary file 1 [file Presentation_1.ppt]

## Slide 1
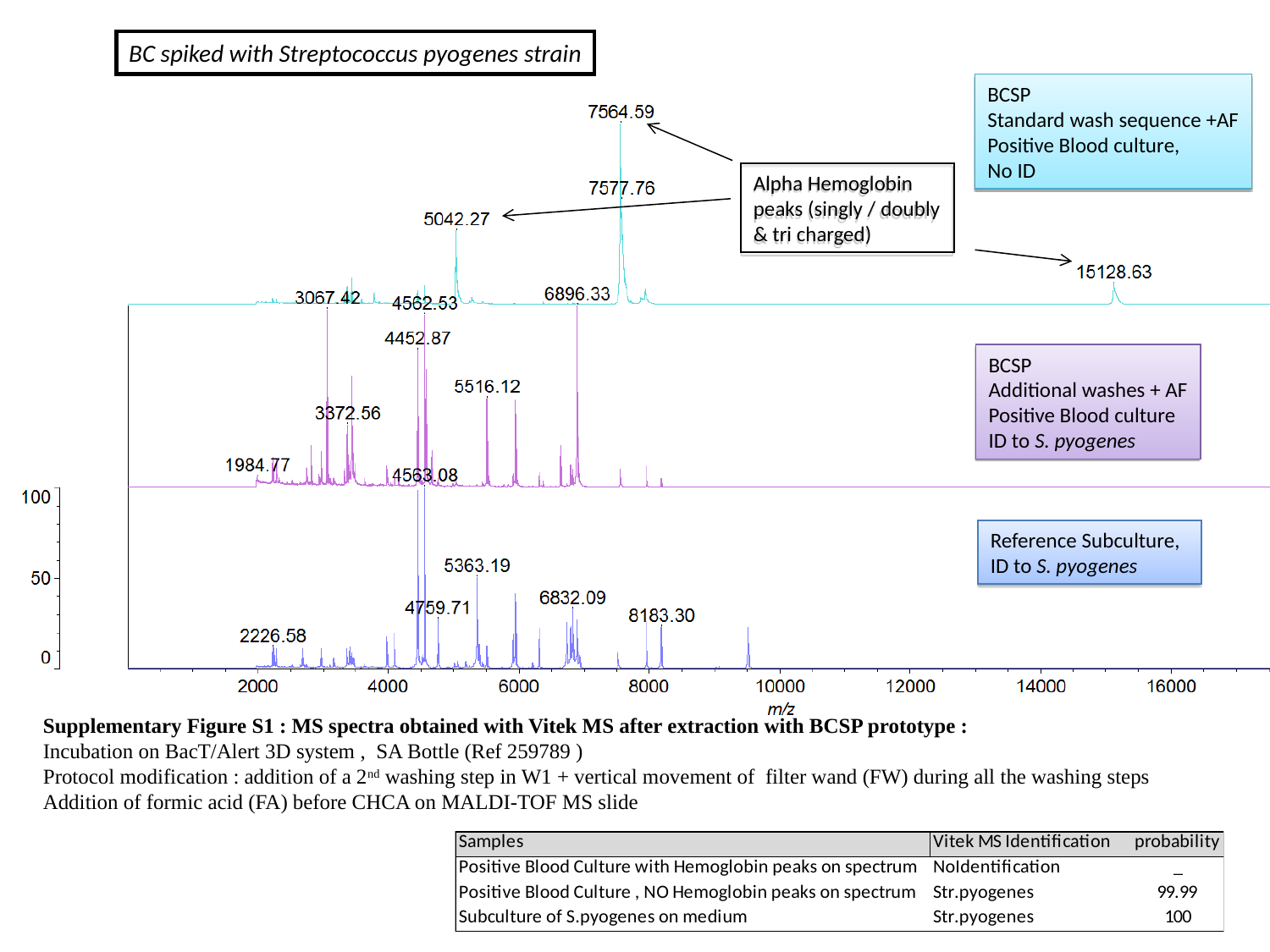

BC spiked with Streptococcus pyogenes strain
BCSP
Standard wash sequence +AF
Positive Blood culture,
No ID
Alpha Hemoglobin peaks (singly / doubly & tri charged)
BCSP
Additional washes + AF
Positive Blood culture
ID to S. pyogenes
Reference Subculture,
ID to S. pyogenes
Supplementary Figure S1 : MS spectra obtained with Vitek MS after extraction with BCSP prototype :
Incubation on BacT/Alert 3D system , SA Bottle (Ref 259789 )
Protocol modification : addition of a 2nd washing step in W1 + vertical movement of filter wand (FW) during all the washing steps
Addition of formic acid (FA) before CHCA on MALDI-TOF MS slide
