# Supplementary material for: An Automated Sample Preparation Instrument to Accelerate Positive Blood Cultures Microbial Identification by MALDI-TOF Mass Spectrometry (Vitek®MS)
Source: Front Microbiol. 2018 May 15;9:911. doi: 10.3389/fmicb.2018.00911 (PMC5962758; doi:10.3389/fmicb.2018.00911)
Supplement: Supplementary file 2 [file Data_Sheet_1.DOC]

# Supplementary Data

## The Filter Wand Extraction & Spotting principle

The FW is manufactured from cylindrical molded polystyrene (Polystyrene Crystal, Atofina) by an injection molding process (Figure 4 right). The upper part of the FW body has a 7mm diameter and reduced to 3.6mm on the tip to fit over the circumference of the Vitek®MS slide spot (2.8mm). The FW pipette is 90mm long, which allows enough volume to hold all the aspirated liquids during the process. The molded FW disposable extremity is manually sealed with a 0.45µm filter membrane (Pall, SuporR) using a solvent based in-house process. In brief, the assembling of the FW is made by soaking the tip in toluene for a few seconds to soften the polystyrene walls and manually applying the filter membrane. After removing excess membrane, polyester fibers are tamped inside the FW to structurally support the membrane during vacuum steps. Additionally, a 30 mm column of 300µm size glass beads are added inside the FW body in order to draw and maintain blood components away from the membrane during the processing steps. A 30° angle was selected at the tip of the FW to facilitate membrane shape. After assembly, the effective filtration diameter is 2.3mm and the membrane has a domed shape to provide efficient contact between the tip and the MALDI slide during automated spotting step.

## The extraction disposable strip and rack description

The extraction disposable strip was developed by designing a 3D printed support (Objet 30, Stratasys, Resin VerowhitePlus) with 5 hole positions in which standard 2mL round bottom tubes are inserted (Simport). Each tube is prefilled and contained (from left to right) as described in Figure 4 (left) :

Well 1 : Empty, BC sample

Well 2 : Selective Lysis buffer (800µL)

Well 3 : Wash buffer 1 (1.4mL)

Well 4 : Wash buffer 1 (1.4mL)

Well 5 : Wash buffer 2 (1.4mL)

The mobile extraction rack (Figure 3) has 12 positions and is made of machined stainless steel to ensure position accuracy of the FW and pipetting tips in the 3 axis (XYZ). After loading the extraction strips in the rack, one 1.2mL pipetting tip (Sartorius, Optifit) and one FW are inserted in the holes of 2 separate spring loading bars. The rack spring loaded bars allows automatic plugging of the FWs and pipetting tips without needing precision servo-motors in the instrument Z axis. The FW has a flange on the upper end which is used as a reference to ensure sufficient accuracy in XY directions during MALDI spotting. The vacuum lines of the 12 positions are connected to the FWs flange surface using standard V-ring rubber seal. Vacuum connection is automatically done when the FWs are plugged and locked with the specific gripper incorporated to the mechanical support moved by the YZ arms. The rack is equipped with a waste bin to collect the used FWs and pipetting tips during the process. The FWs and pipette tips are ejected into the waste bin by YZ arms movements to open the grippers above the rack waste into the instrument .

## The Instrument prototype

The MALDI spotter instrument (Figure 2) is 78 cm long x 55 cm wide 76 cm high, weighs 50 kg and runs on 240V AC power. It communicates with computer-controlled software through an Ethernet cable. The prototype is controlled by custom software developed using Labview code (National Instrument).

The architecture is based on 2 nested arms (Oriental motors) to ensure compactness and rigidity of the mechanical structure during the insertion and ejection of the disposables from/to the rack and during spotting on the MALDI slide. The 2 arms allow manipulation of the pipetting tips and FWs during all the extraction and spotting steps. The 12 pipet tip gripper positions are connected through 0.5mm inner diameter FEP tubing to an e-line 8 channels pipette (1200µL, Sartorius) in order to transfer and mix the lysis buffer with sample (well 1). The e-line pipette is customized to activate the pre-programmed pipetting sequence directly from the computer.

The 12 FW gripper positions are connected through 24 miniature 3-way solenoids (SMC) and a 1-L glass bottle ballast to a vacuum pump (KNF, Laboport Standard) permitting -950 mbar relative pressure with sufficient liquid flow when 12 FWs are used simultaneously. The outlet of the pump is equipped with an HEPA filter to avoid risk of laboratory contamination..

One Vitek®MS slide is loaded on a specific heater block which is placed on a 2 axis platform allowing movement of the MALDI slide to all 48-spot positions during the microorganisms transfer with FW,imaging, CHCA matrix and FA dispensing. This same 2-axis platform also holds a 96 tip rack (Tecan, 1-10µL carbon tips), FA and CHCA standard 0.5mL Tubes (bioMerieux, Marcy l’Etoile) and a small waste bin to collect used 10µL tips., An Air Displacement Pipetor (Cavro ADP, Tecan) is incorporated on the right side of the vertical Z axis arm to automatically dispense 1µL of CHCA matrix and FA on the Vitek®MS slide. The XY arms are configured to collect and eject pipetting tips and aspirate and dispense MALDI reagents. Only one 10µL-tip is used to dispense sequentially 4 x 1µL drops of FA or CHCA matrix after FW spotting.

An imaging module was developed and fixed to the back of the instrument in order to check the quality of deposited biomass during FW spotting and FA and CHCA dispensing and drying. This sub-system is based on a color CMOS camera (Basler acA2500-14gm, 2590x1942 pixels, 1/2.5”) associated with a video objective (P/N NAV1-61453 Navitar adapter-tube is associated with P/N NAV1-61446 Navitar body-tube for a total magnification of 1.2 and a depth of focus of 0.1 mm) and neutral white LED illuminator (P/N NAV1-40028, Navitar) allowing coaxial imaging of each spot after FW biomass transfer. To control the correct matrix crystallization with spotted biomass after CHCA reagent drying, an additional image is made with a second side illumination (a white LED Thin Tspot1, TLP Vision) placed at 30mm of the center of the slide and with a 30° inclination. This sub-system is designed to image a field of 4775 x 3626 µm with a resolution of about 1.8µm /pixel and with a working distance of 92mm. Each image, including XY axis displacements, is taken within 2 seconds per spot position for two illuminations which means 1mn40sec to image a full Vitek®MS slide.
