# Supplementary material for: An Automated Sample Preparation Instrument to Accelerate Positive Blood Cultures Microbial Identification by MALDI-TOF Mass Spectrometry (Vitek®MS)
Source: Front Microbiol. 2018 May 15;9:911. doi: 10.3389/fmicb.2018.00911 (PMC5962758; doi:10.3389/fmicb.2018.00911)
Supplement: Supplementary file 3 [file Data_Sheet_2.DOC]

Table S1. Results per Gram-negative species for strain-inoculated bottles incubated in BacT/Alert® 3D and BacT/Alert VIRTUO systems and identified using direct automated procedure (Blood Culture Sample Preparation (BCSP) prototype and identification by Vitek®MS system).

Depending of the species, 1 or 3 strains have been tested (3 strains for the most prevalent ones).

-: not tested; ID: identification; DSC: discrepant identification.

|  | **Reference ID from BC subculture + Vitek®MS** | | | **BCSP Prototype + Vitek®MS** | | | | | |
| --- | --- | --- | --- | --- | --- | --- | --- | --- | --- |
|  |  |  |  | **BacT/Alert 3D®, SA Bottles** | | | **BacT/Alert Virtuo®, FA Plus bottles** | | |
| 1 strain per species tested | Number of correct ID on total ID (%) | Number of no ID on total ID (%) | Number of DSC on total ID (%) | Number of correct ID on total ID (%) | Number of no ID on total ID (%) | Number of DSC on total ID (%) | Number of correct ID on total ID (%) | Number of no ID on total ID (%) | Number of DSC on total ID (%) |
| *Acinetobacter baumannii* | 2/2  (100%) | / | / | 4/4  (100%) | / | / | - | - | - |
| *Acinetobacter lwoffii* | 2/2  (100%) | / | / | 3/3 (100%) | / | / | - | - | - |
| *Bacteroides fragilis* | 2/2  (100%) | / | / | 4/4 (100%) | / | / | - | - | - |
| *Bacteroides vulgatus* | 4/4 (100%) | / | / | 8/8 (100%) | / | / | - | - | - |
| *Campylobacter coli* | 2/2 (100%) | / | / | 4/4 (100%) | / | / | - | - | - |
| *Citrobacter freundii* | 2/2 (100%) | / | / | 4/4 (100%) | / | */* | - | - | - |
| *Citrobacter koseri* | 6/6 (100%) | / | / | 12/12 (100%) | / | */* | 3/4 (75%) | 1/4  (25%) | / |
| *Enterobacter aerogenes* | 6/6 (100%) | / | / | 12/12 (100%) | / | */* | 3/4 (75%) | / | 1/4  (25%) |
| *Enterobacter cloacae complexe* | 5/6 (83%) | 1/6 (17%) | / | 11/12 (92%) | 1/12 (8%) | */* | 1/4 (25%) | 3/4 (75%) | / |
| *Escherichia coli* | 6/6 (100%) | / | / | 12/12 (100%) | / | / | 4/4 (100%) | / | / |
| *Haemophilus haemolyticus* | 1/4 (25%) | / | 33/4 (75%) | **0/8 (0%)** | 7/8 (88%) | *1/8 (12%)* | - | - | - |
| *Haemophilus influenzae* | 2/2 (100%) | / | / | 4/4 (100%) | / | / | - | - | - |
| *Klebsiella oxytoca* | 6/6 (100%) | / | / | 12/12 (100%) | / | / | 4/4 (100%) | / | / |
| *Klebsiella pneumoniae* | 6/6 (100%) | / | / | 11/12 (100%) | / | / | 4/4 (100%) | / | / |
| *Moraxella catarrhalis* | 2/2 (100%) | / | / | 4/4 (100%) | / | / | / | / | / |
| *Morganella morganii* | 6/6 (100%) | / | / | 12/12 (100%) | / | / | 4/4 (100%) | / | / |
| *Neisseria gonorrhoeae* | 6/6 (100%) | / | / | 0/22 (0%) | 10/12 (83%) | 2/12 (17%) | - | - | - |
| *Neisseria meningitidis* | 8/8 (100%) | / | / | 6/16 (38%) | 10/16 (62%) | / | - | - | - |
| *Proteus mirabilis* | 6/6 (100%) | / | / | 12/12 (100%) | / | / | 4/4 (100%) | / | / |
| *Proteus vulgaris* | 2/2 (100%) | / | / | 4/4 (100%) | / | / | - | - | - |
| *Providencia stuartii* | 2/2 (100%) | / | / | 4/4 (100%) | / | / | - | - | - |
| *Pseudomonas aeruginosa* | 6/6 (100%) | / | / | 12/12 (100%) | / | / | 4/4 (100%) | / | / |
| *Salmonella enterica serovar Enteritidis* | 2/2 (100%) | / | / | 4/4 (100%) | / | / | - | - | - |
| *Serratia liquefaciens* | 2/2 (100%) | / | / | 4/4 (100%) | / | / | - | - | - |
| *Serratia marcescens* | 2/2 (100%) | / | / | 4/4 (100%) | / | / | - | - | - |
| *Stenotrophomonas maltophilia* | 2/2 (100%) | / | / | 3/4 (75%) | 1/4 (25%) | / | - | - | - |
| *Yersinia enterocolitica* | 2/2 (100%) | / | / | 4/4 (100%) | / | / | - | - | - |
| **Gram negative bacteria** | 100/104 (96%) | 1/104 (1%) | 3/104 (3%) | 174/206 (84%) | 29/206 (15%) | 3/206 (1%) | 31/36 (86%) | 4/36 (11%) | 1/36 (3%) |

**Table S2. Results per Gram-positive and yeast species for strain-inoculated bottles incubated in BacT/Alert® 3D and BacT/Alert VIRTUO systems and identified using direct automated procedure (Blood Culture Sample Preparation (BCSP) prototype and identification by Vitek®MS system).**

Depending of the species, 1 or 3 strains have been tested (3 strains for the most prevalent ones).

**-: not tested; ID: identification; DSC: discrepant identification**

|  | **Reference ID from BC subculture + Vitek®MS** | | | | | **BCSP Prototype + Vitek®MS** | | | | | | | | |
| --- | --- | --- | --- | --- | --- | --- | --- | --- | --- | --- | --- | --- | --- | --- |
|  |  |  | |  | | **BacT/Alert 3D®, SA Bottles** | | | **BacT/Alert Virtuo®, FA Plus bottles** | | | | | |
| 1 strain per species tested | Number of correct ID on total ID (%) | | Number of no ID on total ID (%) | | Number of DSC on total ID (%) | Number of correct ID on total ID (%) | Number of no ID on total ID (%) | Number of DSC on total ID (%) | Number of correct ID on total ID (%) | Number of no ID on total ID (%) | | Number of DSC on total ID (%) | |  |
| *Bacillus simplex* | 2/2  (100%) | | / | | / | 4/4 (100%) | / | / | - | | - | | - | |
| *Clostridium difficile* | - | | - | | - | 4/4  (100%) | / | / | - | | - | | - | |
| *Clostridium perfringens* | 2/2  (100%) | | / | | / | 4/4  (100%) | / | / | - | | - | | - | |
| *Corynebacterium striatum* | 6/6 (100%) | | / | | / | 8/12 (67%) | 4/12 (33%) |  | - | | - | | - | |
| *Enterococcus avium* | 2/2  (100%) | | / | | / | 4/4  (100%) | / | / | - | | - | | - | |
| *Enterococcus faecalis* | 6/6 (100%) | | / | | / | 12/12 (100%) | / | / | 4/4  (100%) | | / | | / | |
| *Enterococcus faecium* | 6/6 (100%) | | / | | / | 11/12 (92%) | 1/12 (8%) | / | 3/4 (75%) | | 1/4 (25%) | | / | |
| *Enterococcus gallinarum* | 2/2  (100%) | | / | | / | 4/4  (100%) | / | / | - | | - | | - | |
| *Listeria monocytogenes* | 2/2  (100%) | | / | | / | 4/4  (100%) | / | / | - | | - | | - | |
| *Micrococcus luteus/lylae* | 2/2  (100%) | | / | | / | 4/4  (100%) | / | / | - | | - | | - | |
| *Propionibacterium acnes* | 4 | | / | | / | 0/8 (0%) | 8/8 (100%) | / | - | | - | | - | |
| *Staphylococcus aureus* | 6/6 (100%) | | / | | / | 12/12 (100%) | / | / | 3/4 (75%) | | 1/4 (25%) | | / | |
| *Staphylococcus capitis* | 2/2 (100%) | | / | | / | 4/4  (100%) | / | / | - | | - | | - | |
| *Staphylococcus epidermidis* | 6/6 (100%) | | / | | / | 12/12 (100%) | / | / | 1/4 (25%) | | 3/4 (75%) | | / | |
| *Staphylococcus haemolyticus* | 2/2 (100%) | | / | | / | 4/4  (100%) | / | / | 4/4  (100%) | | / | | / | |
| *Staphylococcus hominis* | 2/2 (100%) | | / | | / | 4/4  (100%) | / | / | 4/4  (100%) | | / | | / | |
| *Staphylococcus lugdunensis* | 2/2 (100%) | | / | | / | 3/4 (75%) | 1/4 (25%) | / | - | | - | | - | |
| *Staphylococcus saprophyticus* | 2/2 (100%) | | / | | / | 4/4  (100%) | / | / | - | | - | | - | |
| *Staphylococcus warneri* | 2/2 (100%) | | / | | / | 4/4  (100%) | / | / | - | | - | | - | |
| *Streptococcus agalactiae* | 6/6 (100%) | | / | | / | 10/12 (83%) | 2/12 (17%) | / | 4/4  (100%) | | / | | / | |
| *Streptococcus anginosus* | 6/6 (100%) | | / | | / | 11/12 (92%) | 1/12 (8%) | / | - | | - | | - | |
| *Streptococcus constellatus* | 6/6 (100%) | | / | | / | 12/12 (100%) | / | / | - | | - | | - | |
| *Streptococcus infantarius subsp. Coli* | 2/2 (100%) | | / | | / | 3/4 (75%) | 1/4 (25%) | / | - | | - | | - | |
| *Streptococcus intermedius* | 4/6 (68%) | | 1/6 (16%) | | 1/6 (16%) | 12/12 (100%) | / | / | 4/4  (100%) | | / | | / | |
| *Streptococcus oralis* | 2/2 (100%) | | / | | / | 3/4 (75%) | 1/4 (25%) | / | - | | - | | - | |
| *Streptococcus pneumoniae* | 6/6 (100%) | | / | | / | 11/12 (92%) | 1/12 (8%) | / | 4/4  (100%) | | / | | / | |
| *Streptococcus pyogenes* | 6/6 (100%) | | / | | / | 8/12 (67%) | 4/12 (33%) | / | - | | - | | - | |
| ***Gram positive*** | 96/98 **(98%)** | | 1/98 **(1%)** | | 1/98 **(1%)** | 176/200 **(88%)** | 24/200 **(12%)** | 0/200 **(0%)** | 31/36 **(86%)** | | 5/36 **(14%)** | | 0 (0%) | |
| *Candida albicans* | 6/6 (100%) | | / | | / | 12/12 (100%) | / | / | 1/4 (25%) | | 3/4 (75%) | | / | |
| *Candida glabrata* | 6/6 (100%) | | / | | / | 4 | / | / | - | | - | | - | |
| *Candida krusei* | 2/2 (100%) | | / | | / | 4 | / | / | 4/4  (100%) | | / | | / | |
| *Candida parapsilosis* | 2/2 (100%) | | / | | / | 4 | / | / | 4/4  (100%) | | / | | / | |
| *Candida tropicalis* | 2/2 (100%) | | / | | / | 12 | / | / | 3/4 (75%) | | 1/4 (25%) | | / | |
| ***Yeast*** | 18/18 **(100%)** | | 0 | | 0 | 36/36 **(100%)** | 0 | 0 | 12/16 **(75%)** | | 4/16 **(25%)** | | 0 | |

**Table S3.** Comparison of percentage of identification rate: Chi-square test

|  | **BacT/Alert *versus* Reference** | **Virtuo *versus* Reference** | **BacT *versus* Virtuo** |
| --- | --- | --- | --- |
| Gram-negative species | p_value = 0.003 | p_value = 0.03 | p_value=0.83 |
| Gram-positive species | p_value = 0.004 | p_value = 0.007 | p_value=0.78 |
| Yeast species | p_value = 1 | p_value = 0.02 | p_value = 0.002 |
| Overall ID Rate | p_value < 0.001 | p_value < 0.001 | p_value=0.41 |

**Table S4. Number of strains tested per species**

| **Gram-negative Species** | **Number of strains tested per species** | **Gram –positive Species** | **Number of strains tested per species** |
| --- | --- | --- | --- |
| | *Escherichia coli* | | --- | | *Pseudomonas aeruginosa* | | *Klebsiella pneumoniae* | | *Proteus mirabilis* | | *Enterobacter cloacae Cpx* | | *Enterobacter aerogenes* | | *Klebsiella oxytoca* | | *Morganella morganii* | | *Citrobacter koseri* | | *Stenotrophomonas maltophilia* | | *Citrobacter freundii* | | *Serratia marcescens* | | *Acinetobacter baumannii* | | *Campylobacter coli* | | *Bacteroides fragilis* | | *Bacteroides vulgatus* | | *Haemophilus influenzae* | | *Haemophilus haemolyticus* | | *Moraxella catarrhalis* | | *Yersinia enterocolitica* | | *Salmonella enterica ser Enteritidis* | | *Neisseria meningitidis* | | *Neisseria gonorrhoeae* | | *Bacillus simplex* | | *Proteus vulgaris* | | *Providencia stuartii* | | *Serratia liquefaciens* | | *Acinetobacter lwoffii* | | | 3 | | --- | | 3 | | 3 | | 3 | | 3 | | 3 | | 3 | | 3 | | 3 | | 1 | | 1 | | 1 | | 1 | | 1 | | 1 | | 2 | | 1 | | 2 | | 1 | | 1 | | 1 | | 4 | | 3 | | 1 | | 1 | | 1 | | 1 | | 1 | | | *Staphylococcus aureus* |  | | --- | --- | | *Staphylococcus epidermidis* |  | | *Enterococcus faecalis* |  | | *Streptococcus pneumoniae* |  | | *Streptococcus anginosus* |  | | *Streptococcus constellatus* |  | | *Streptococcus intermedius* |  | | *Streptococcus agalactiae* |  | | *Enterococcus faecium* |  | | *Staphylococcus haemolyticus* |  | | *Staphylococcus hominis* |  | | *Streptococcus pyogenes* |  | | *Clostridium difficile* |  | | *Clostridium perfringens* |  | | *Propionibacterium acnes* |  | | *Staphylococcus saprophyticus* |  | | *Corynebacterium striatum* |  | | *Listeria monocytogenes* |  | | *Micrococcus luteus* |  | | *Staphylococcus capitis* |  | | *Streptococcus oralis* |  | | *Staphylococcus lugdunensis* |  | | *Staphylococcus warneri* |  | | *Streptococcus infantarius subs Coli* |  | | *Enterococcus gallinarum* |  | | *Enterococcus avium* |  | |  |  | |  |  | |  |  | | | 3 | | --- | | 3 | | 3 | | 3 | | 3 | | 3 | | 3 | | 3 | | 3 | | 1 | | 1 | | 3 | | 1 | | 1 | | 2 | | 1 | | 3 | | 1 | | 1 | | 1 | | 1  1 | | 1 | | 3 | | 1 | | 1 | |

| **Yeast Species** | **Number of strains tested per species** |
| --- | --- |
| *Candida albicans*  *Candida tropicalis*  *Candida glabrata*  *Candida parapsilosis*  *Candida krusei* | 3  3  1  1  1 |
